# Role of the dorsolateral prefrontal cortex in processing temporal anomalies retained in working memory

**DOI:** 10.3389/fnbeh.2024.1494227

**Published:** 2024-11-11

**Authors:** Pierandrea Mirino, Alessandro Quaglieri, Gabriele Scozia, Sara Mercuri, Alessandro Alessi, Cecilia Guariglia, Anna Maria Giannini, Fabrizio Doricchi, Anna Pecchinenda

**Affiliations:** ^1^Department of Psychology, Sapienza University of Rome, Rome, Italy; ^2^AI2life s.r.l., Innovative Start-Up, ISTC-CNR Spin-Off, Via Sebino, Rome, Italy; ^3^Department of Human and Social Science, Mercatorum University, Rome, Italy; ^4^IRCCS Santa Lucia Foundation, Rome, Italy; ^5^Department of Medicine and Health Science “Vincenzo Tiberio”, Campobasso, Italy

**Keywords:** attention, visuo-spatial memory, timing, Time Squares Sequence, DLPFC (dorsolateral prefrontal cortex)

## Abstract

**Introduction:**

Time is a crucial abstract construct, allowing us to perceive the duration of events. Working memory (WM) plays an important role in manipulating and storing the different features of environmental stimuli, including temporal features. Different brain structures, including the dorsolateral prefrontal cortex, are involved in time processing.

**Methods:**

Here we investigated the functional aspects of time processing by using functional near-infrared spectroscopy (fNIRS) to assess changes in DLPFC activity. A modified version of the “Times Squares Sequences” (TSS) task was used, in which participants are required to match sequences of squares that have fixed or variable durations.

**Results:**

Findings showed that the DLPFC activates when information necessary for later comparison needs to be maintained online, as is common in visuo-spatial WM tasks. Importantly, the DLPFC deactivates when a temporal anomaly is detected.

**Discussion:**

This deactivation occurs because the temporal anomaly does not require ongoing maintenance for later comparison, thus demanding fewer cognitive resources from the DLPFC. This seemingly counterintuitive effect can be attributed to the temporal aspects being irrelevant to the primary task goals. This finding highlights the crucial role of implicit temporal interference and establishes a strong connection between timing and executive cognitive processes.

## 1 Introduction

Any given stimulus present in nature has many different features (e.g., quantity, shape, color) and depending on current goals, processing this information calls upon different cognitive functions (e.g., attention, memory, decision making, working memory, perception, etc.). Working memory (WM) functions have a fundamental role in processing, manipulating, and storing information, allowing to achieve pre-set objectives and plan goal-directed behaviors (Baddeley and Hitch, 1974). One of the most salient features processed by WM is time (Ferrandez et al., 2003; Pan and Luo, 2012; Üstün et al., 2017).

Time represents an abstract construct that adds order and coherence to the events we experience every day (Üstün et al., 2017). The working memory contribution to time processing allows establishing a beginning and an ending to the stimulus duration and making judgments on time intervals (Üstün et al., 2017).

The ability to compute time duration relies on the contribution of different brain circuits, involving both cortical and subcortical structures (Liu et al., 2008; Manohar and Husain, 2016a, b; Wu et al., 2010). More specifically, in a fMRI study, Üstün et al. (2017) observed higher activations in both parietal and frontal areas, insula, supplementary motor area, inferior olive nucleus, cerebellum, and basal ganglia. In particular, the dorsolateral prefrontal cortex (DLPFC) plays a key role in working memory functions, such as maintaining online information related to task goals, information storage, as well as in updating and manipulating information. In addition, it seems to be involved in top-down modulation of selective attention as it allows to boost or inhibit attentional resources and neural activity towards relevant information depending on our goals (Ungerleider, 2000; Pecchinenda et al., 2015; Sdoia et al., 2019; Zanto et al., 2011). Together with the parietal lobe (particularly the intraparietal sulcus), it is involved in top-down modulation of selective attention as it allows to boost, inhibit and shift attentional resources and neural activity towards relevant information depending on our goals (Ungerleider, 2000; Ferrandez et al., 2003; Lewis and Miall, 2006; Zanto et al., 2011; Üstün et al., 2017; Sdoia et al., 2019; Zanto et al., 2011; Pecchinenda et al., 2021). Moreover, the supplementary motor area (SMA), which projects to the basal ganglia, is also involved in sensory and motor tasks requiring timekeeping and information updating, especially under increased cognitive load (Courtney et al., 1997_;_ Üstün et al., 2017; Wittmann et al., 2010; Harrington et al., 1998). The insular cortex, with its posterior part, encodes the passage of time, the frontal part reproduces time intervals, and the cerebellum acts as an “internal clock” in tasks involving learning and sensory-motor coordination (Spencer et al., 2003). Although various brain structures contribute to time detection, no specific circuitry or activation priority map can be established. However, fMRI studies indicate that processing tactile stimulations with different temporal characteristics involves the activation of cerebellar and brainstem structures, particularly the “olive-cerebellar complex” (Liu et al., 2008; Wu et al., 2010; Xu et al., 2006). This finding is in keeping with previous models on the information-processing (IP) of interval timing such as the pacemaker-accumulator models, which originally proposed that interval timing entails three stages – clock, memory, and decision (Creelman, 1962; Treisman, 1963). Before, during and after the perception of time intervals, the pacemaker provides pulses that are gated into an accumulator by attention, and then working memory is necessary in judging the entity of the accumulated pulses, comparing it in long-term memory and then making a decision on the time perceived.

Taken together existing literature points to the DLPFC crucial role in working memory and its control over maintaining, organizing, and manipulating information. However, whereas there is good evidence on the role of the DLPFC in processing temporal characteristics when explicitly required, there is insufficient evidence for implicit temporal processing. Our goal is to assess the role of the dorsolateral prefrontal cortex (DLPFC) in visuo-spatial working memory tasks, specifically when participants are unaware of the temporal variation of the stimuli presented.

To investigate whether individuals can detect this type of temporal variations, here defined as a temporal “anomaly,” we used the “Times Squares Sequences” (TSS), a new computerized working memory task, in which visuo-spatial stimuli of fixed or variable duration are presented during the encoding phase and then during the recognition phase. A behavioral study by Mirino et al. (2023) using this task showed that participants’ performance is affected by these temporal anomalies. This effect has been attributed to the fact that, when we memorize a sequence of events, adding a temporal variable can affect encoding. Indeed, there is evidence that simultaneous processing of visual–spatial stimuli has a lower attentional and mnemonic load, as the stimuli can be viewed as a single pattern (Lupo et al., 2018). In contrast, different presentation times interfere with encoding and recognition processes because a variable sequence of stimuli involves a higher cognitive load as, in addition to processing the single stimulus, the time between stimuli in the entire sequence must also be encoded. This higher cognitive load is reflected in poorer performance.

Building on this evidence, here, we report a study aimed at investigating the anatomo-functional aspects of time processing by using functional near-infrared spectroscopy (fNIRS). fNIRS is a noninvasive, optical imaging technique that records the hemodynamic changes of Oxy-hemoglobin, Deoxy-hemoglobin, and Total Hemoglobin of a given tissue.

Previous studies have demonstrated that fNIRS provides reliable data for DLPFC activity as it can consistently detect changes in oxygenated and deoxygenated hemoglobin levels across trials and sessions and that this signal significantly correlates with fMRI signals (Fishburn et al., 2014; Pinti et al., 2020). Indeed, recording and analyzing the activity of the DLPFC during visuospatial tasks using fNIRS is a research method that is gaining progressive validity and reliability thanks to the non-invasive nature and relatively high temporal resolution of this technique.

In the present study, we measured changes in the activity of the DLPFC involved in processing implicit temporal anomalies during the visuo-spatial working memory task entailed by the “Times Squares Sequences” (TSS). More specifically, we assessed the changes underlying the behavioral effects of longer RTs when there is a mismatch between S1 and S2 where S2 has a time anomaly as observed in Mirino et al. (2023). We expected differences in DLPFC activity between conditions, especially during the recognition phase (H1) with a higher activation of DLPFC related to WM load (H2).

## 2 Materials and methods

### 2.1 Participants

We conducted a sensitivity power analysis to assess the minimum effect size we could detect with our sample. This showed that with 45 participants we could detect an effect size of 0.36, corresponding to a medium-large effect size compared to the 0.25 medium effect size reported by Mirino et al. (2023). Participants who are not right-handed have uncorrected vision impairments, a history of psychiatric or neurological disorders (such as epilepsy or stroke), or are currently receiving pharmacological treatment that may affect cognitive, or motor function will be excluded from the study. Based on these criteria fifty-one volunteers took part in the study, 6 participants were excluded because they did not respond to more than 25% of the trials. This resulted in 45 participants (18 Males and 27 Females), aged between 18 and 39 years (Mean = 23.36; SD = 4.50), years in school (Mean = 14.78; SD = 2.43). The test was administered between June and December 2022. The experimental protocol was approved by the Ethics Committee of the Department of Psychology, Sapienza University of Rome (protocol number 0000272, 17.02.2022).

### 2.2 Materials

#### 2.2.1 Task—Time Squares Sequences (TSS)

**Figure 1:**
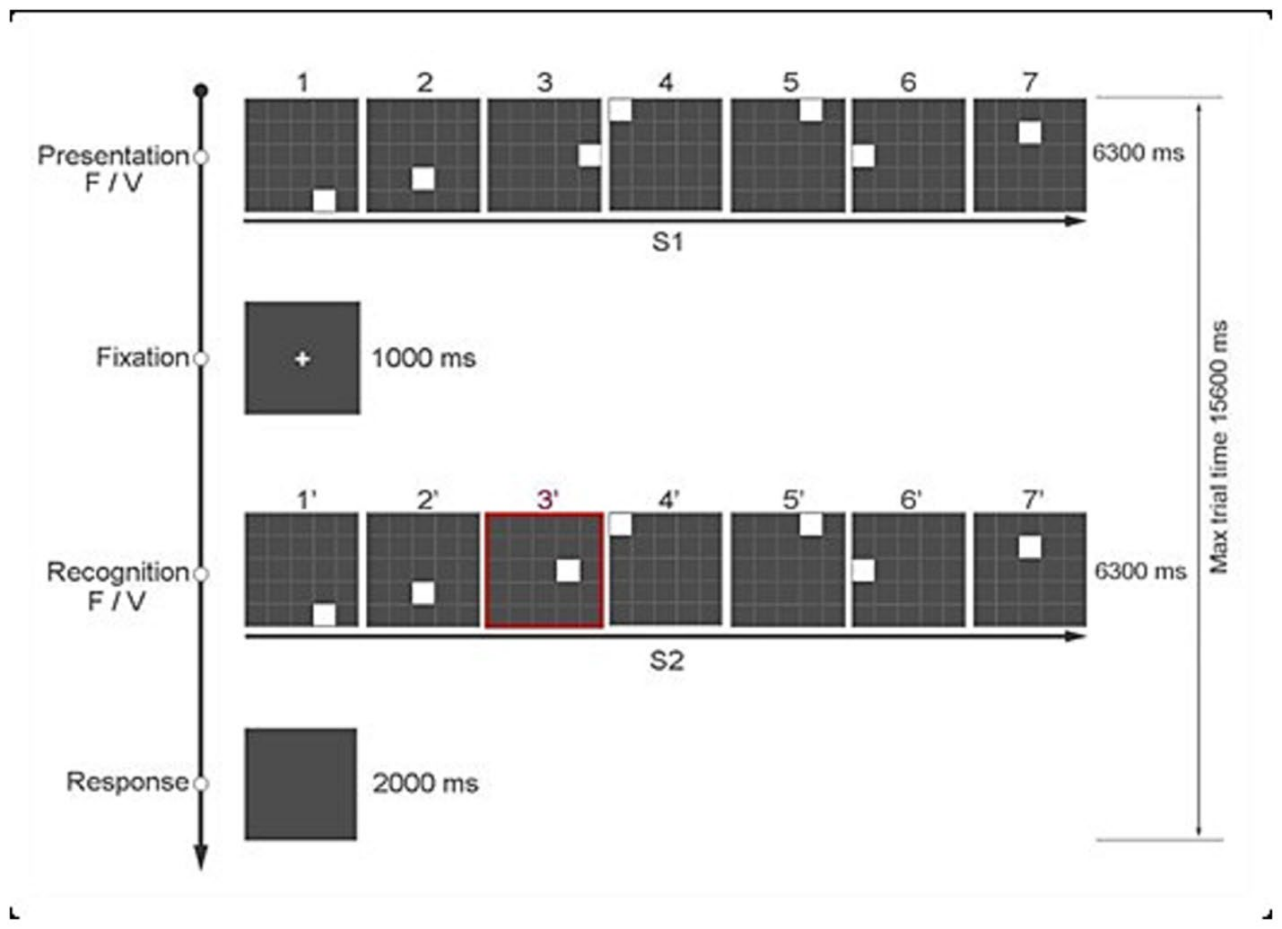
The Time Squares Sequences task (TSS).

The Time Squares Sequences Task (TSS, Figure 1) previously described in Mirino et al. (2023) was modified to adapt it for the fNIRS study (i.e., to minimize overlap of the hemodynamic response). Compared to the original TSS, which consisted of 2 blocks of 120 trials, in the present paradigm we used a single block of 120 trials presented in random order, with a jittered intertrial interval (ITI) ranging from 3,400 msec to 6,400 msec, varying in steps of 300 msec.

Each trial consists of two sequences – S1 and S2 – and participants decide whether S2 matches S1. Each sequence has 7 white squares (S1), presented one at a time. That is, when one square disappears, the next one immediately follows. Each white square is presented for a variable duration (i.e., 300–1,500 msec) on a 5 by 5 grid of gray squares. The grey squares are delimited by white lines. The total duration of each sequence (S1) is 6,300 msec, followed by an interval of 1,000 msec, after which the second sequence of squares (S2) is presented. Unbeknownst to the participants, each sequence of 7 squares in S1 and S2 could either be regular – that is, each square is presented for 900 msec (i.e., fixed sequence) – or it could have a temporal anomaly, as each square is presented for a different duration (i.e., variable sequence). An example of a variable time sequence is one in which the 7 squares are presented for 300 msec, 1,200 msec, 1,500 msec, 1,200 msec, 600 msec 300 msec 1,200 msec, respectively. In addition, the position on the grid of one of the 7 squares in the two sequences (S1 and S2) could be the same or different. The experimental manipulations of spatial position (task-relevant) and temporal duration (task-irrelevant) for the 7 white squares in S1 and S2 result in 4 different conditions of 30 trials each:

1. S1: Fixed Sequence – S2: Fixed Sequence (FF)
2. S1: Fixed Sequence – S2: Variable Sequence (FV)
3. S1: Variable Sequence – S2: Fixed Sequence (VF)
4. S1: Variable Sequence – S2: Variable Sequence (VV).

Each participant responded by pressing one of two keys of the keyboard based on whether the positions of the 7 squares in S1 and S2 were the same (key “z”) or different (key “m”). The keys were labeled accordingly and could only be pressed at the end of S2 up to a maximum of 2,000 msec. To indicate to the participants when they could answer, the screen slightly changed to gray. We presented the task using Open Science Tools Ltd. (Peirce et al., 2019). Figure 1 shows the sequence of events in a typical trial.

#### 2.2.2 Functional near-infrared spectroscopy (fNIRS)

fNIRS data were collected by using the NIRSport2 mobile system (NIRx Medical Technologies, LLC, New York, USA), which has 8 emitters and 8 detectors, the inter-optode distance for each channel was set to 3 cm. During data collection, the sampling frequency of the device was set to 8.71 Hz, employing two distinct wavelengths (i.e., 760 and 850 nm) for the acquisition of intensity data. The brain activity and the subsequent changes in blood flow alter the concentration of oxy- and deoxy-hemoglobin in the recorded tissue, which in turn differentially changes the absorption of light at different wavelengths due to the two forms of hemoglobin with different optical absorption profiles. Changes in hemoglobin can be recovered from fNIRS measurements at multiple wavelengths via the modified Beer–Lambert law (Huppert et al., 2009). For the fNIRS, optodes positioning was based on the methodology employed in previous research (Zimeo et al., 2018; Schommartz et al., 2021). To ensure accurate placement of the optodes, cross-verification with anatomical landmark atlases such as AAL2 (Tzourio-Mazoyer et al., 2002; Rolls et al., 2015), Brodmann (Rorden and Brett, 2000), and Juelich (Eickhoff et al., 2007) using the fOLD Optodes’ Location Decider (Zimeo et al., 2018) was used. This verification confirmed that the predefined cortical regions of interest (ROIs) corresponded to the specified channels on the left and right sides, ensuring accurate coverage of the DLPFC. Consistent with the inherent limitations of this approach, the methodology does not incorporate subject-specific structural MRI scans or a 3-D digitizer (Selb et al., 2014; Tsuzuki and Dan, 2014), limiting the exact spatial co-registration of cortical coordinates to specific MNI coordinates. Given the broad scope of DLPFC activity captured by fNIRS (Fishburn et al., 2014; Choe et al., 2016; Vassena et al., 2014) and the inherent spatial resolution of fNIRS, finer anatomical localization is not affordable. Therefore, this solution allows effective measurement of DLPFC activity with the resolution expected from fNIRS technology.

**Figure 2:**
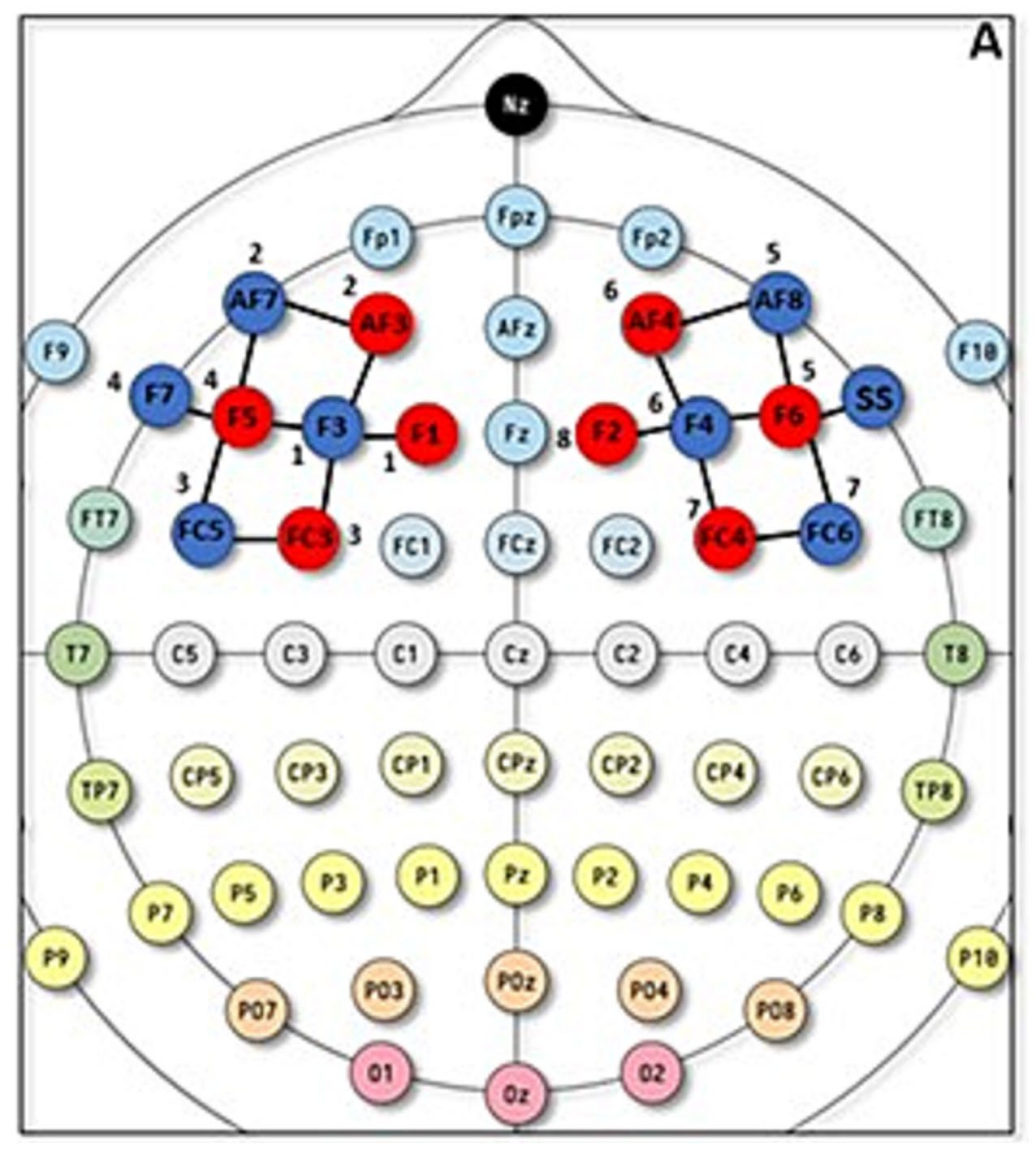
fNIRS optodes positioning. A = fNIRS optode setup for the left and right DLPFC according to the 5–10 system.

Specifically, based on Schommartz et al. (2021), we assessed oxygenation of the prefrontal cortical region, corresponding to the dLPFC (Brodmann’s area 8, 9, 46). Specifically, fNIRS optode setup for the left and right dlPFC (i.e., emitters I and detectors) were positioned according to the international 10/20 and the exact positioning provided by Schommartz et al., 2021 (Figure 2), specifically, into channels 1 (F1-F3), 2 (AF3-F1), 4 (FC3-F3), 6 (F5-F3), and 7 (F5-AF7) on the left side and channels 11 (F6-AF8), 13 (F6-F4), 15 (AF4-F4), 17 (FC4-F4), and 18 (F2-F4) on the right side. In addition, eight 8-mm channels were used, and an extra silicon photodiode detector was split into 8 groups of dual detectors, with each group surrounding a light source at an 8-mm distance. The eight short-channel detectors were located on two hemispheres, with 4 on the left DLPFC and 4 on the right DLPFC. Figure 2 A shows the exact optode placement in our study.

### 2.3 Procedure

After providing informed consent, participants completed the pre-screening interview (see exclusion criteria in the Participants section), and the Corsi test (Spinnler, 1987). Only participants with a visuospatial memory span of 4 (Miller, 1991) were invited to complete the task. First head circumference was measured to choose the proper fNIRS headcap and prepare the DLPFC optode montage. After a signal quality check, participants were asked to sit in a comfortable position and follow the instructions on a computer screen that would lead them through the task. After completing the set-up stage and a short practice task, participants completed the Time Square Sequences (TSS) for an average total duration of 37 min. Participants performed a matching task where they decided whether the square-stimuli of two sequences (S1 and S2) had the same or different positions. Even if the time duration of the two sequences presented at encoding and at recognition is always the same, the two can differ as they can have a fixed duration, fixed and variable duration, or variable and fixed duration, respectively. The presentation order of the 4 different conditions was randomized between subjects, and the overall duration of the experiment was about 60 min.

#### 2.3.1 Data pre-processing

For each condition, we computed: (a) an Accuracy Index (AI) as (HIT +CR) / (total trial number/4) where 4 is the number of conditions (FF, FV, VF, VV), which can vary from 0 to 1, and (b) an overall Accuracy Index (AI) for each single subject. In addition, (c) the mean Response Time (RTs) computed for Hit, CR, Fa, and MISS in each condition, and (d) an overall Response Time for a single subject. We also screened data for outliers using 2 SDs as a cut-off (Miller, 1991). This resulted in 6 outliers (i.e., the slowest individuals) for response time.

**Figure 3:**
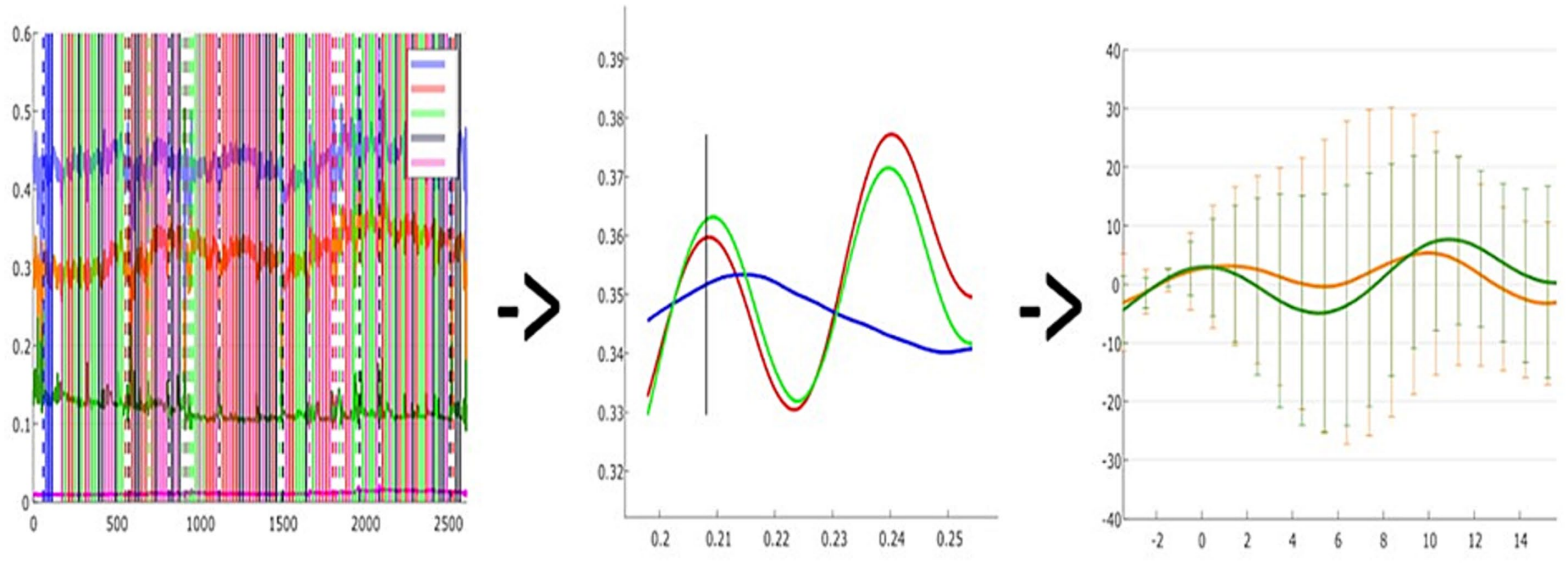
A visual representation of data processing steps: A = Raw signal recording of a single subject; B = Hemodynamic response function plot of a single subject, consisting of oxy (RED) - deoxy (BLUE) - Total (GREEN) hemoglobin; C = Grand-averaged oxy-hemoglobin changes in a given task condition (e.g., two channels in FF condition).

The fNIRS data signals were pre-processed using the open-source software analysis toolbox Homer3 based on Matlab. The raw signal was first inspected by all the authors in order to manually reject invalid and noisy trials from the analysis, this strategy was adopted also to manually exclude the time where explicit artifacts occurred. The second step was the conversion of the raw signal to Optical Densities (ODs), which does not require the definition of any parameter. Then, based on the OD signal, a motion artifact function identified for each channel changes greater than std-tresh (50.0) or amp_thresh (5.0), which were considered an artifact, and a segment of data around that time point is marked. In addition, we run a Motion correction Spline function; by performing a cubic spline correction of the motion artifacts the algorithm follows the procedure described by Scholkmann et al. (2010). After these corrections were done, we ran a bandpass filter on time course data, with a high-pass filter of 0.01 and a low-pass filter value of 0.50. At this point, we applied the Modified Beer–Lambert Law to convert ODs data to hemodynamic concentrations. Here partial path length factors had been defined for each wavelength, but convention is becoming to set ppf = 1 and to not divide by the source-detector separation such that the resultant concentration is in units of Molar mm. Although this procedure is often used in the literature (Strangman et al., 2003; Yücel et al., 2021), there is no specific reference for it. After obtaining the Hemodynamic Response Function, a block averaging on concentration data was computed for each experimental condition (FF, FV, VF, VV). More specifically, a time window of −3.4 and 15.6 s from the condition was used. The statistical analysis of Hemodynamic Response Function (i.e., HRF) data was conducted on SPSS Statistics 26 after exporting the matrices of HRF mean values of each subject (see Figure 3).

### 2.4 Data analysis

With respect to the behavioral measures, we analyzed Response Time (RT) data with SPSS Statistics 26 (IBM, Armonk, NY), with an ANOVA with Temporal Condition (4: FF, FV, VF, VV) as a within-subject factor. To exclude age-related effects, and consistent with the data on prefrontal cortex development (e.g., Casey et al., 2000; Gogtay and Thompson, 2010), we conducted additional and separately 2×4 ANOVA analyses considering RT and Accuracy as within-groups and Age-groups as between-subjects factors and gender as a covariate. To divide the sample, we used the median with a value of 23.36, thus obtaining the first group of *N* = 25 with an age between 18 and 23 years and the second group of *N* = 20 with an age between 24 and 39 years. As no significant effects due to age and gender were found, we conducted the subsequent analysis on a single group. We reported the results in the supplementary material for clarity.

Furthermore, correlation analysis and the trade-off between RT and Accuracy Index were computed in each condition (see Supplementary Figures S1–S4 and Supplementary Table S3).

The distributions of all data were verified for normality. Greenhouse–Geisser corrections were applied when Mauchly’s test indicated a violation of the sphericity assumption. Post-hoc tests for temporal conditions were Bonferroni corrected for multiple comparisons.

To analyze fNIRS data we used two different strategies. First, based on the results of preliminary analyses (see the Supplementary material for a detailed description) we focused the current ANOVA on Oxyhemoglobin. Second, to better map the behavioral findings, and understand the underlying neural mechanisms, we performed two separate ANOVAs one for the vertical and the other for the horizontal channel. The two ANOVAs mainly focused on the differences observed in the behavioral analysis and on the channels most sensitive to the experimental manipulations (and the most spatial corresponding with the DLPFC) during recording sessions. Each ANOVA had Experimental Condition (4: FF-FV-VF-VV), and Hemispheres (2: Left–Right) as within-subject factors.

## 3 Results

### 3.1 Behavioral results

Results for RTs averaged across blocks showed a main effect of Temporal Condition (F (2,3; 102,17) = 19.29, *p* < 0.001; η_p2_ 0.305). Bonferroni corrected post-hoc comparisons showed faster RTs in FF (*M* = 578.09, SD = 173.08) than in FV (*M* = 660.60, SD = 172.58) (delta, *M* = − 83.00, SE = 15.00, *p* < 0.001), and VV (*M* = 657.34, SD = 196.04) (delta, *M* = −79.00, SE = 12.00, *p* < 0.001), indicating that participants were faster when a temporal anomaly is not present (i.e., the time presentation of stimuli is equal in both the encoding and recognition phase).

**Figure 4:**
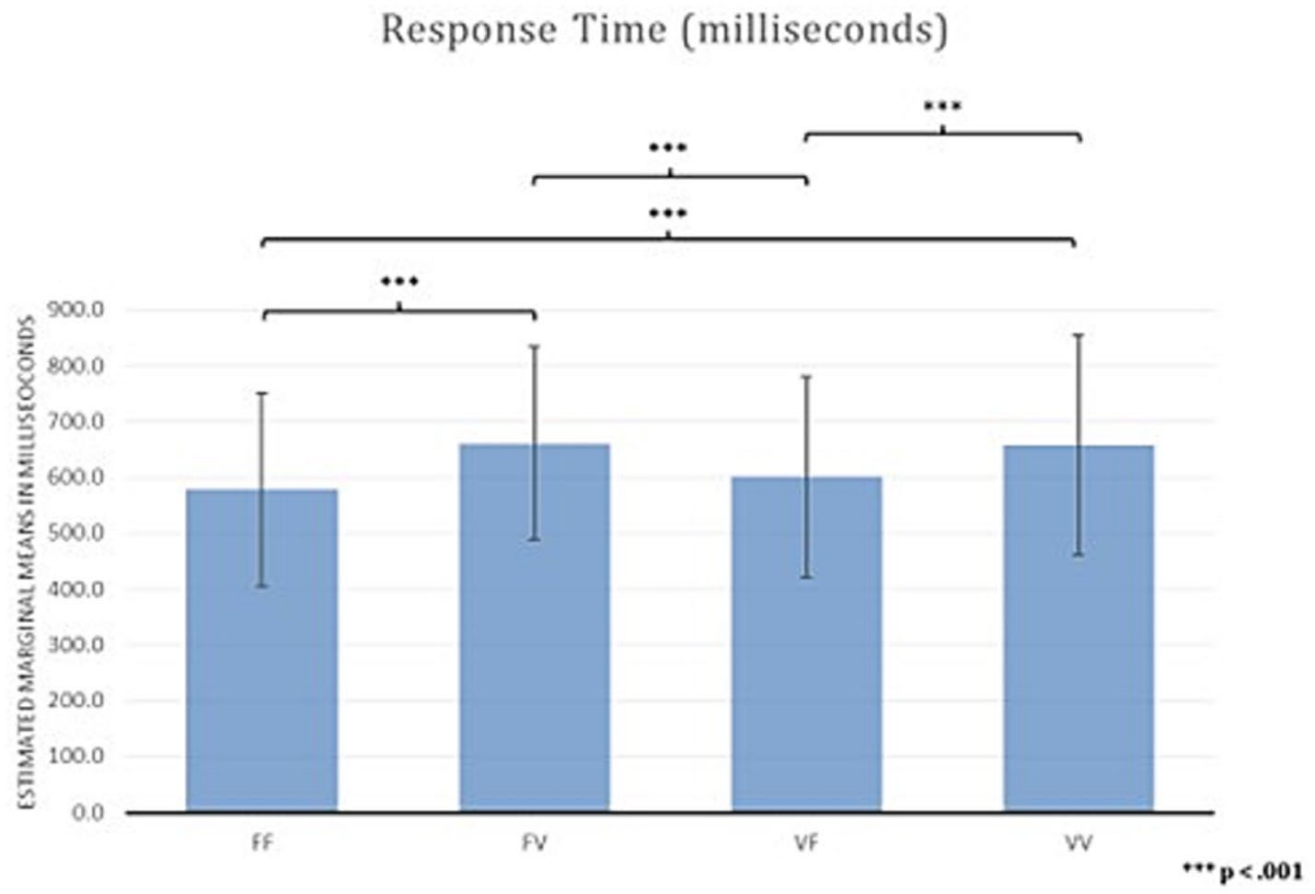
Response time of Time Squares Sequences task.

There were also faster RTs in VF (*M* = 601.00, SD = 179.00) compared to FV (*M* = 660.60, SD = 172.58) (delta, *M* = −60.00, SE = 12.00, *p* < 0.001) and to VV (*M* = 657.34, SD = 196.04) (delta, *M* = −56.00, SE = 12.00, *p* < 0.001) (Figure 4), indicating that participants were faster only when the temporal anomaly is present in the encoding phase. In contrast, RTs did not differ between FF and VF (*M* = −23.00, SE = 10.00, *p* = 0.165), and between FV and VV (delta, *M* = 3.00, SE = 17.00, *p* > 0.99).

Results for the Accuracy Index showed no statistically significant main effect of Temporal Condition (F (3;132) = 0.728, *p* = 0.537; η_p2_ = 0.016). Correlation analysis did not show any statistically significant results.

For all the descriptive statistics and correlation analysis results see Supplementary Tables S1–S3.

### 3.2 fNIRS results

The behavioral results are in line with the findings reported in Mirino et al. (2023), showing that a change in the stimuli temporal characteristic (i.e., an anomaly) affected RTs only when the temporal anomaly occurred in the sequence presented in the recognition phase. This suggests a mechanism, operating after the encoding phase, for monitoring the progress of events over time. To assess the neural underpinnings of this effect, we analyzed changes in the activity of the DLPFC during the task.

**Figure 5:**
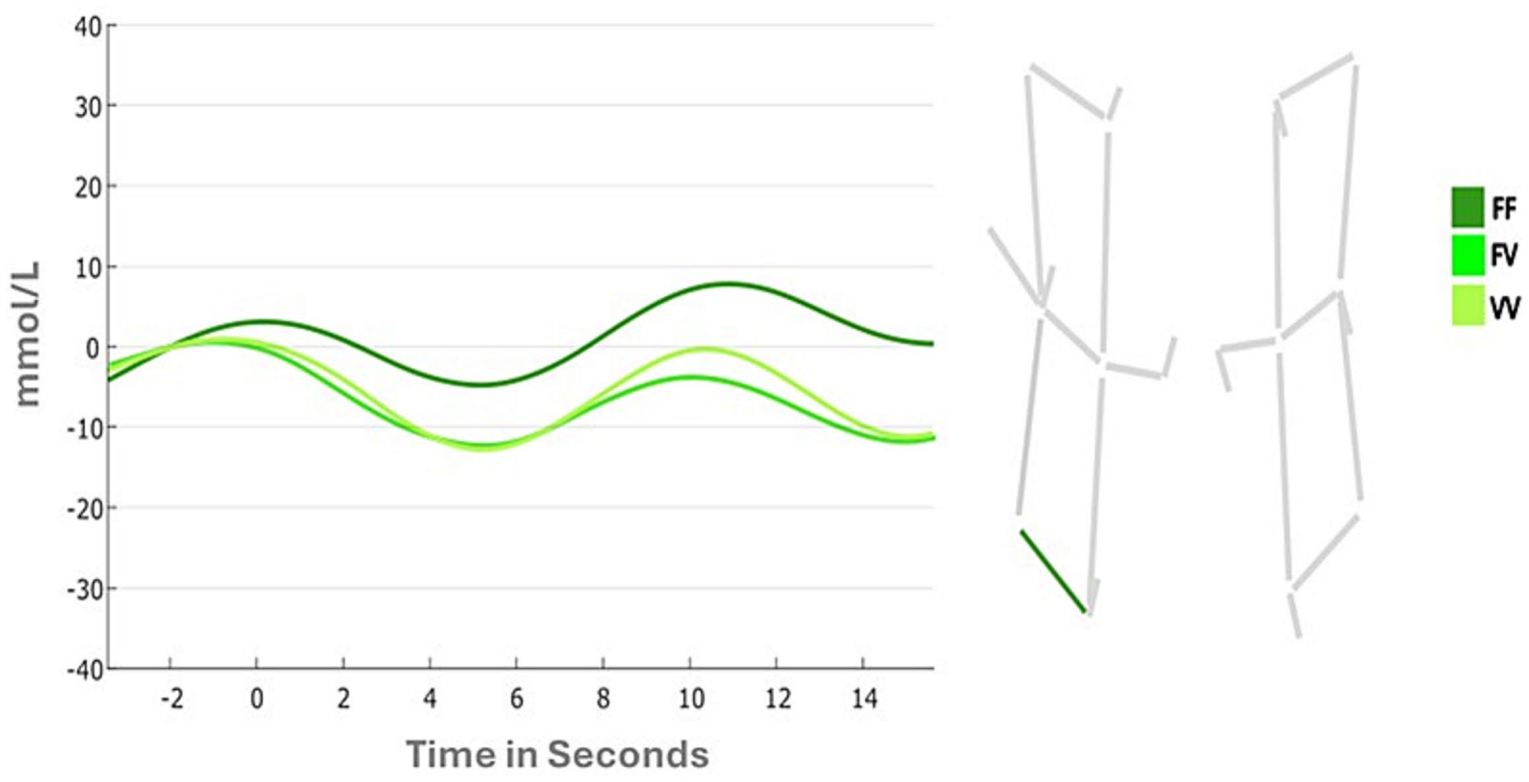
Changes in oxyhemoglobin between task conditions (i.e., FF, FV, VV) in horizontal channels.

Results of the first 4 (Condition) by 2 (Hemispheres) ANOVA for the horizontal channel showed no significant main effect of Condition (*F*_(1,41)_ = 2.0242; *p* = 0.11; ƞ_p2_ = 0.047). The main effect of Hemispheres was marginally significant (F _(1,41)_ = 3.722; *p* = 0.06; ƞ_p2_ = 0.083), while the Condition by Hemispheres interaction was significant (*F*_(3,123)_ = 3.293; *p* = 0.02; ƞ_p2_ = 0.074). Follow-up analyses showed that the interaction was due to different patterns of DLPFC activation in the left hemispheres across the 4 conditions (FF: 0.138 mmoL/L; FV: −0.749 mmoL/L; VV: −0.605 mmoL/L, Figure 5), and different pattern of DLPFC activation between the two hemispheres during the FF condition (Left: 0.138 mmoL/L; Right: −0.902 mmoL/L). In particular, Bonferroni comparisons suggest that the Left hemisphere is more activated during the whole task and that in this hemisphere there was a gradient of activation across different conditions: the DLPFC was more activated in the FF condition rather than the VV condition, and in the FV it was observed the lowest activation among all conditions.

**Figure 6:**
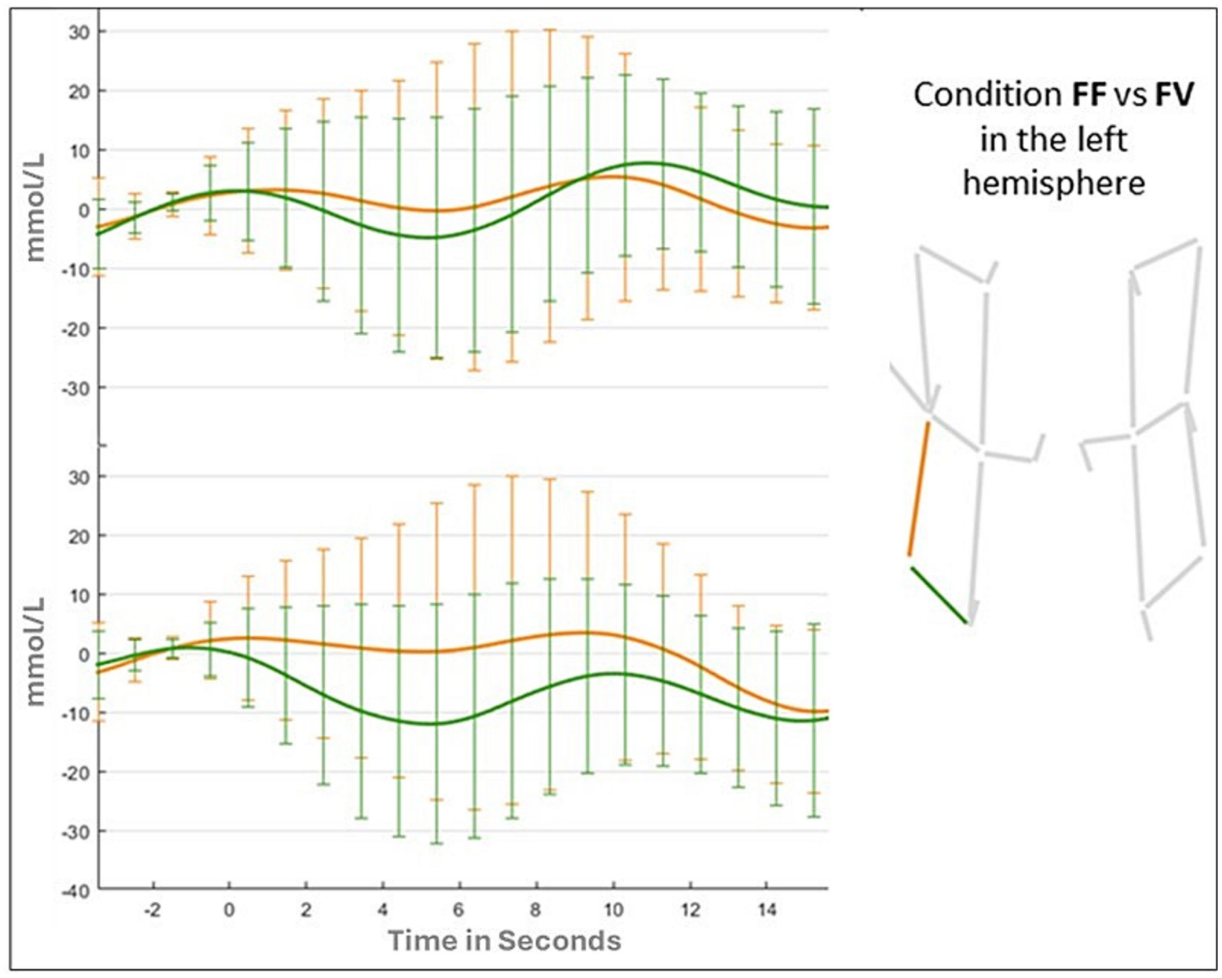
Oxyhemoglobin value in the left hemisphere for FF and FV conditions.

**Figure 7:**
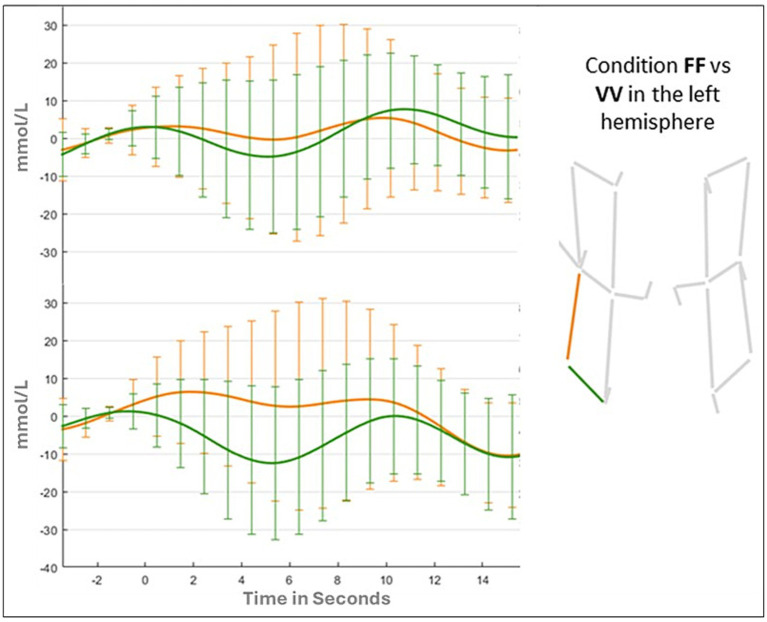
Oxyhemoglobin value in the left hemisphere for FF and VV conditions.

Results of the ANOVA for the vertical channel showed a significant main effect of Hemispheres (F _(1,41)_ = 6.322; *p* = 0.01; ƞ_p2_ = 0.015), due to a greater activation for the left hemisphere (Left: 0.139 mmoL/L; Right: −0.774 mmoL/L). The main effect of Condition (F _(1,41)_ = 0.543; *p* = 0.65; ƞ_p2_ = 0.013) and the Condition by Hemispheres were not statistically significant (F _(3,123)_ = 0.419; *p* = 0.73; ƞ_p2_ = 0.013). Changes in oxy-hemoglobin for the two channels (vertical and horizontal) in FF, FV, and VV conditions are shown in Figures 6, 7.

## 4 Discussion

We investigated the involvement of the DLPFC during a visuo-spatial working memory task with stimuli characterized by temporal anomalies. We expected a difference in participants’ performance between conditions, especially during the recognition phase (S2 phase), and a different activation of the DLPFC related to the working memory load entailed by the different experimental conditions. In line with Mirino et al. (2023) behavioral findings showed that the temporal anomaly in the second sequence slows down participants’ responses. This happens when the temporal anomaly occurs in the sequence of stimuli presented in the recognition phase (i.e., the condition FV) and when it was present in both encoding and recognition phase (i.e., the condition VV), suggesting that in these two conditions, there is an increased WM load. This account is in line with a study of Blalock and Clegg (2010), where the variation in participants’ performance was investigated by using a task with simultaneous or delayed sequences of stimuli. Their findings showed greater performance accuracy in the condition with simultaneous presentation, which according to the authors was due to the fact that, when a sequence of events is stored, adding a time variable affects the encoding phase. Indeed, the literature has shown that simultaneous processing of visuospatial stimuli has a lower load on attention and memory, as the stimuli can be viewed as a single pattern (Lupo et al., 2018). In addition, important insights on this effect also come from a study by Brown (2006) on the types of interference in dual tasks (temporal vs. non-temporal). The author showed that bi-directional interference was present for tasks that tap the putative “central executive” component of working memory. Tasks such as motor tracking or visual search were not affected by concurrent timing, as they did not interfere with each other. Instead, bidirectional interference seems to be more pronounced in tasks like random-number generation, sequential reasoning, mental arithmetic, proofreading, and searching working memory (Brown, 2006). The findings of the present study add to this evidence by showing that task-irrelevant temporal anomalies interfere with the central executive component of WM, which worsens the participant’s performance in a visuo-spatial sequential matching task. Previous studies have highlighted the crucial role of the DLPFC in processing information during working memory tasks. For instance, Guevara et al. (2018) observed higher absolute power of slow EEG bands in the DLPFC during visuospatial working memory tasks, associated with the increased difficulty of cognitive tasks and with the inhibitory processes (Zarjam et al., 2012; Engle et al., 1999a, 1999b; Kane and Engle, 2003).

In addition, in line with our first hypothesis (H1), findings from the functional near-infrared spectroscopy (fNIRS) showed that the brain activity of the DLPFC is reduced during task conditions in which the temporal anomalies are in the recognition phase (i.e., FV) or are present in both the encoding and recognition phases (i.e., VV). Interestingly, and in contrast to what was predicted, we observed a reduction of Oxyhemoglobin in our region of interest, suggesting a de-activation of this area. Therefore, the presence of temporal anomalies not only delays response times but also reduces DLPFC activation.

The behavioral and physiological effects observed when the temporal anomaly was present in the recognition phase (i.e., FV and VV) but not when the anomaly occurred in the encoding phase (i.e., VF) are driven by the fact that when a temporal anomaly is present in the S1, the participant encoded the anomaly with the other stimuli characteristics. In contrast, when the anomalies occurred after encoding, the temporal variation is not necessary to judge the two sequences as the “same” or “different” (S1-S2), also considering that in the VV condition, the S1 and S2 temporal anomalies never correspond.

Importantly, the role of the DLPFC is crucial for comparing sequences in the FF condition. As task instructions require that participants encode the spatial position of stimuli in a grid whereas the temporal characteristic of stimuli is task-irrelevant, our results showed that the DLPFC is activated in the FF and VF conditions to maintain online task-relevant information. This might be used for later comparison (i.e., as usual in visuo-spatial working memory tasks). This entails that when a temporal anomaly is detected—possibly by sub-cortical structures – then a de-activation of the DLPFC is observed as the temporal anomaly needs not to be maintained online for later comparison. Accordingly, less cognitive resources are required for the DLPFC. This account for this counterintuitive effect relies on the task irrelevant temporal features. When a temporal anomaly is present during the encoding phase, it is probably encoded as part of the stimulus but is not considered relevant because it is not necessary for the comparison during the recognition phase. However, when the anomaly is present in both the presentation and recognition phases, the two temporal variations are different. In this case, the first anomaly is encoded with the stimulus, while the second, being different, is detected as an anomaly. The same dynamics occur when the anomaly is present only in the recognition phase and not in the encoding phase.

In this last case, the anomaly, not having been detected previously, acts as an additional cognitive load, as a salient event that shifts attentional focus. Considering the role of the fronto-parietal network in attentional resources, we can speculate that the anomalies present in our paradigm might involve the activation of other areas, leading to the concurrent deactivation of the DLPFC. This attentional shift and the subsequent refocusing to complete the task can result in increased response times. However, this account is *a posteriori* and future research should further explore the proposed mechanism, incorporating advanced neuroimaging techniques and examining diverse populations to further elucidate the DLPFC functions in temporal processing. Our findings complement existing research on hemispheric differences in the DLPFC’s function during tasks involving temporal sequences. Specifically, the left DLPFC is associated with structured temporal processing (Allman and Meck, 2012; Allman et al., 2014). In our study, its involvement during the recognition of temporal anomalies indicates that this region supports working memory related to temporal aspects, even when such anomalies serve as distractions. The observed deactivation may indicate a redirection of cognitive resources to networks, like the fronto-parietal network, that manage unexpected events, highlighting the left hemisphere’s crucial role in engaging sequential processing resources under increased working memory load and attentional demands.

The fronto-parietal network plays a key role in attentional control and cognitive flexibility, allowing the brain to adapt quickly to unanticipated stimuli. Studies have shown that this network, which includes regions like the DLPFC and the intraparietal sulcus (IPS), helps redirect cognitive resources when facing unexpected or distracting events (Wang et al., 2010). Zanto and Gazzaley (2013) further highlights that the fronto-parietal network functions as a flexible hub of cognitive control, dynamically allocating attention to support the management of increasing working memory load and attentional demands. This mechanism could explain the deactivation observed in the left DLPFC during temporal anomalies, as cognitive resources may shift towards the fronto-parietal network to handle the unexpected stimuli.

Understanding these dynamics can lead to more effective strategies for managing cognitive load and improving performance in various contexts. The importance of the DLPFC in adapting to contextual changes during decision-making is also supported by García-Hernández et al. (1791) and Iribe-Burgos et al. (1779), who found changes in DLPFC EEG activity during reversal learning conditions in a decision-making task. These results converge with previous studies that have highlighted the crucial role of the DLPFC in processing information during working memory tasks.

Finally, future studies should clarify any potential role of the DLPFC in Implicit Sequence Learning (ISL), which entails temporal characteristics as well as the neural correlates of those transient modulations of ISL which appear to be driven by rather more subtle oscillations of brain structures involved in cognitive control (Prutean et al., 2021).

### 4.1 Limitations

The present findings showed the crucial role of implicit interference and established a close link between timing and executive processing. However, considering the sensitivity and signal-to-noise ratio of fNIRS, the present study has several limitations. Future studies could consider both a larger sample size and the implementation of a paradigm with the combined use of EEG to assess the role of inter-individual differences, a more precise electrode montage, and integration of structural magnetic resonance imaging scans or 3D digitizer in fNIRS. The dynamic nature of decision-making and the importance of information updating have also been highlighted by Cortes et al. (1769), who identified distinct EEG patterns for different stages of the decision-making process, particularly during the formation of preferences and outcome evaluation. Future studies could integrate these perspectives for a more comprehensive understanding of the DLPFC’s role in temporal decision-making.

Future studies could consider comparative analyses between different age groups, both to explore whether this ability is automatic or learned through experience and to investigate whether it may be functionally impaired as a result of cognitive impairment (e.g., mild cognitive impairment) or as a result of other pathological conditions. Future studies could consider comparative analyses between different age groups, both to explore whether this ability is automatic or learned through experience and to investigate whether it may be functionally impaired as a result of cognitive impairment (e.g., mild cognitive impairment) or other pathological conditions. Additionally, it would be valuable to investigate this aspect by comparing gender, constituting larger groups to base the comparison on. This is motivated by the literature, which consistently reports on sex differences in time perception (Hancock and Rausch, 2010) making it essential to discuss this thoroughly - especially given that the experimental sample was predominantly feminine. However, in our study, we controlled gender by treating it as a covariate, which did not appear to influence the final results.

Indeed, other brain areas of particular interest in time perception, not considered in this study, should be investigated in our results. Previous studies have demonstrated the influence of other brain regions in identifying temporal variations of stimuli [e.g., the inferior olive, cerebellum, and supplementary motor area (Xu et al., 2006)]. However, the focus of this study was to specifically investigate the contribution of the DLPFC brain region, which has previously been shown to be most involved in working memory functions.

## Data Availability

The raw data supporting the conclusions of this article will be made available by the authors, without undue reservation.

## TOP guidelines statement

No part of the study procedures or analyses was preregistered prior to the research being conducted. We report how we determined our sample size, all data exclusions (if any), all inclusion/exclusion criteria, whether inclusion/exclusion criteria were established prior to data analysis, all manipulations, and all measures in the study.
