# Supplementary material for: Role of the dorsolateral prefrontal cortex in processing temporal anomalies retained in working memory
Source: Front Behav Neurosci. 2024 Nov 11;18:1494227. doi: 10.3389/fnbeh.2024.1494227 (PMC11586175; doi:10.3389/fnbeh.2024.1494227)
Supplement: Supplementary file 1 [file Data_Sheet_1.docx]

***Supplementary Material***

# Supplementary Behavioral Data

Results for RTs averaged across blocks showed a main effect of Temporal Condition (F (2,3; 107,79) = 8.86, p < .001; η_p2_ .174). No interaction effect between RT and Age group (F (2,3; 107,79) = .115, p = .916; η_p2_ .003), no interaction effect of RT and Gender (F (2,3; 107,79) = .672, p = .533; ηp2 .016).

Result for Accuracy showed no main effect of Accuracy (F (3; 126,00) = .483, p = .694; η_p2_ .011), no interaction effect between RT and Age group (F (3; 126,00) = .112, p = .953; η_p2_ .003), no interaction effect of RT and Gender (F (3; 126,00) = 1.617, p = .189; ηp2 .037).

# Supplementary Physiological Data

The result of the first 2x2x2x3 Anova with Experimental Condition (FF vs. FV), Hemispheres (Left-Right), Channels (2 channels), Hemodynamic Concentration (O2Hb – HHb – tHb) as within factors pointed-out a significant main effect of Experimental Condition (F (1,41) = 5.025; p = 0.030; ƞp2 = 0.109), showing a minor activation of the bilateral dlPFC during FV condition (-0.453 mmol/l) in contrast to FF condition (-0.113 mmol/l) (Figure 4). The interaction among all factors (Experimental Condition x Hemispheres x Channels x Hemodynamic Concentration was significant (F (2,82) = 3.062; p = 0.013; ƞp2 = 0.099). Bonferroni Post-Hoc showed that the OxyHemoglobin of the vertical channels (i.e., F5, FC5, F6, FC6) was significantly different in the two hemispheres during FF condition (Left 0.160 mmol/l vs. Right -0.575 mmol/l, p = 0.027); the same bilateral significant difference was observed during FV condition (Left -0.031 mmol/l vs. Right -1.045 mmol/l, p < 0.001).

Furthermore, there was a significant difference in the OxyHemoglobin for the left horizontal channel (FC5, FC3) between the experimental condition FF and FV (FF 0.138 mmol/l vs. FV -0.749 mmol/l, p = 0.001); a significant difference between hemispheres (FC5, FC3, FC4, FC6) was observed during FF condition in the horizontal channel (Left 0.138 mmol/l vs. Right -0.902 mmol/l, p < 0.001).

The result of the second 2x2x2x3 Anova with Experimental Condition (FF vs. VV), Hemispheres (Left-Right), Channels (2 channels), Hemodynamic Concentration (O2Hb – HHb – tHb) as within factors highlighted a significant main effect of Channels (F (1,41) = 8.415; p = 0.005; ƞp2 = 0.170), showing that the general activity in horizontal channels is lower than the activity in the vertical ones (-0.337 mmol/l vs. 0.00011 mmol/l). The interaction among all factors (Experimental Condition x Hemispheres x Channels x Hemodynamic Concentration was significant (F (2,82) = 7.412; p = 0.001; ƞp2 = 0.153). Bonferroni Post-Hoc showed that the OxyHemoglobin of the vertical channel was different between hemispheres during FF condition (Left 0.160 mmol/l vs. Right -0.575 mmol/l, p = 0.027); the same bilateral significant difference was observed during VV condition (Left 0.123 mmol/l vs. Right -0.643 mmol/l, p < 0.001).

Moreover, there was a significant difference in the OxyHemoglobin for the left horizontal channel (FC5, FC3) between the experimental condition FF and FV (FF 0.138 mmol/l vs. VV -0.605 mmol/l, p < 0.001); a significant difference between hemispheres (FC5, FC3, FC4, FC6) was observed during FF condition in the horizontal channel (Left 0.138 mmol/l vs. Right -0.902 mmol/l, p < 0.001).

Finally, we have examined the potential age-related effect on the prefrontal cortex development. We run two separate ANOVA, one for each channel (horizontal and vertical), with Experimental Condition (4: FF-FV-VF-VV), and Hemispheres (2: Left-Right) as within-subject factors, and age group as a categorical variable. We did not find any effect of age. Specifically in the analysis of the horizontal channel, we found a significant effect of Experimental condition x Hemispheres interaction (F _(3,120)_ = 3.219; p = 0.02; ƞp2 = 0.074), and a not significant effect of Experimental condition x Hemispheres x Age interaction (F _(3,120)_ = 0.459; p = 0.71; ƞp2 = 0.011). In the analysis of the vertical channel, we found only a significant main effect of Hemispheres (F _(1,40)_ = 6.142; p = 0.01; ƞp2 = 0.133), and not a significant effect of Experimental condition x Hemispheres x Age interaction (F _(3,120)_ = 0.436; p = 0.72; ƞp2 = 0.010).

# Supplementary Figures and Tables

| **Temporal condition** | **Mean** | **Standard deviation** | **Minimum** | **Maximum** |
| --- | --- | --- | --- | --- |
| **RTs_FF** | 0.58 | 0.17 | 0.27 | 0.99 |
| **RTs_FV** | 0.66 | 0.17 | 0.34 | 1.07 |
| **RTs_VF** | 0.60 | 0.18 | 0.29 | 0.99 |
| **RTs_VV** | 0.66 | 0.20 | 0.31 | 1.20 |

Table S1. Descriptive statistics of Response Time (RT in seconds)

| **Temporal condition** | **Mean** | **Standard deviation** | **Minimum** | **Maximum** |
| --- | --- | --- | --- | --- |
| **AI_FF** | 0.60 | 0.13 | 0.20 | 0.90 |
| **AI_FV** | 0.60 | 0.13 | 0.33 | 0.90 |
| **AI_VF** | 0.62 | 0.13 | 0.30 | 0.87 |
| **AI_VV** | 0.61 | 0.11 | 0.37 | 0.87 |

Table S2. Descriptive statistics of Accuracy Index (ACC)

| **Correlation** |  | **ACC_FF** | **ACC_FV** | **ACC_VF** | **ACC_VV** |
| --- | --- | --- | --- | --- | --- |
| **TR_FF** | Pearson | -0.002 | -0.143 | -0.239 | -0.013 |
|  | Sig. | 0.988 | 0.316 | 0.091 | 0.926 |
| **TR_FV** | Pearson | 0.001 | -0.142 | -0.237 | -0.011 |
|  | Sig. | 0.994 | 0.320 | 0.095 | 0.937 |
| **TR_VF** | Pearson | -0.001 | -0.142 | -0.237 | -0.012 |
|  | Sig. | 0.997 | 0.321 | 0.093 | 0.932 |
| **TR_VV** | Pearson | 0.000 | -0.142 | -0.237 | -0.012 |
|  | Sig. | 1.000 | 0.322 | 0.094 | 0.933 |

Table S3. Correlations between RT and ACC


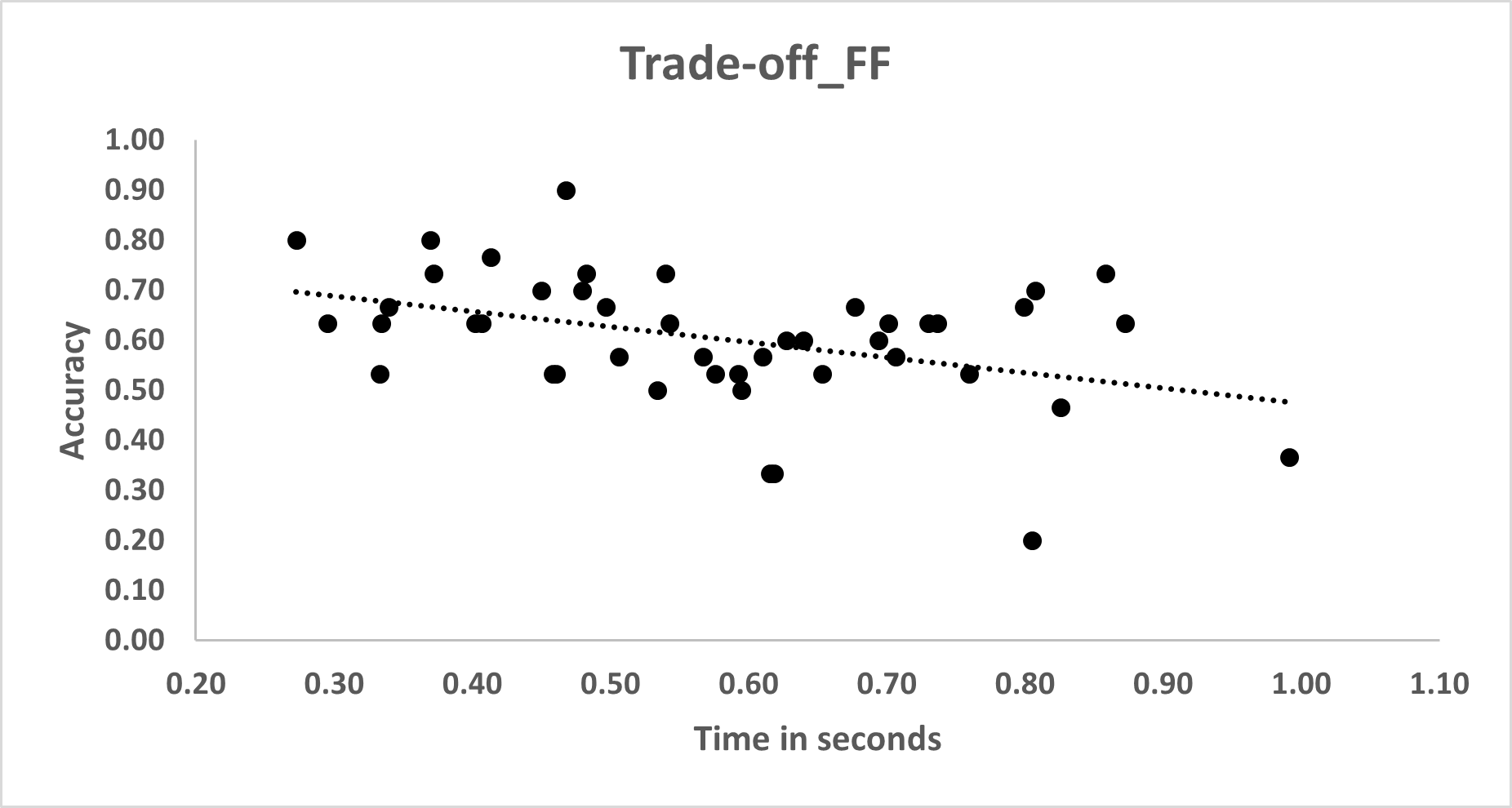


Figure S1. Trade-off in FF condition


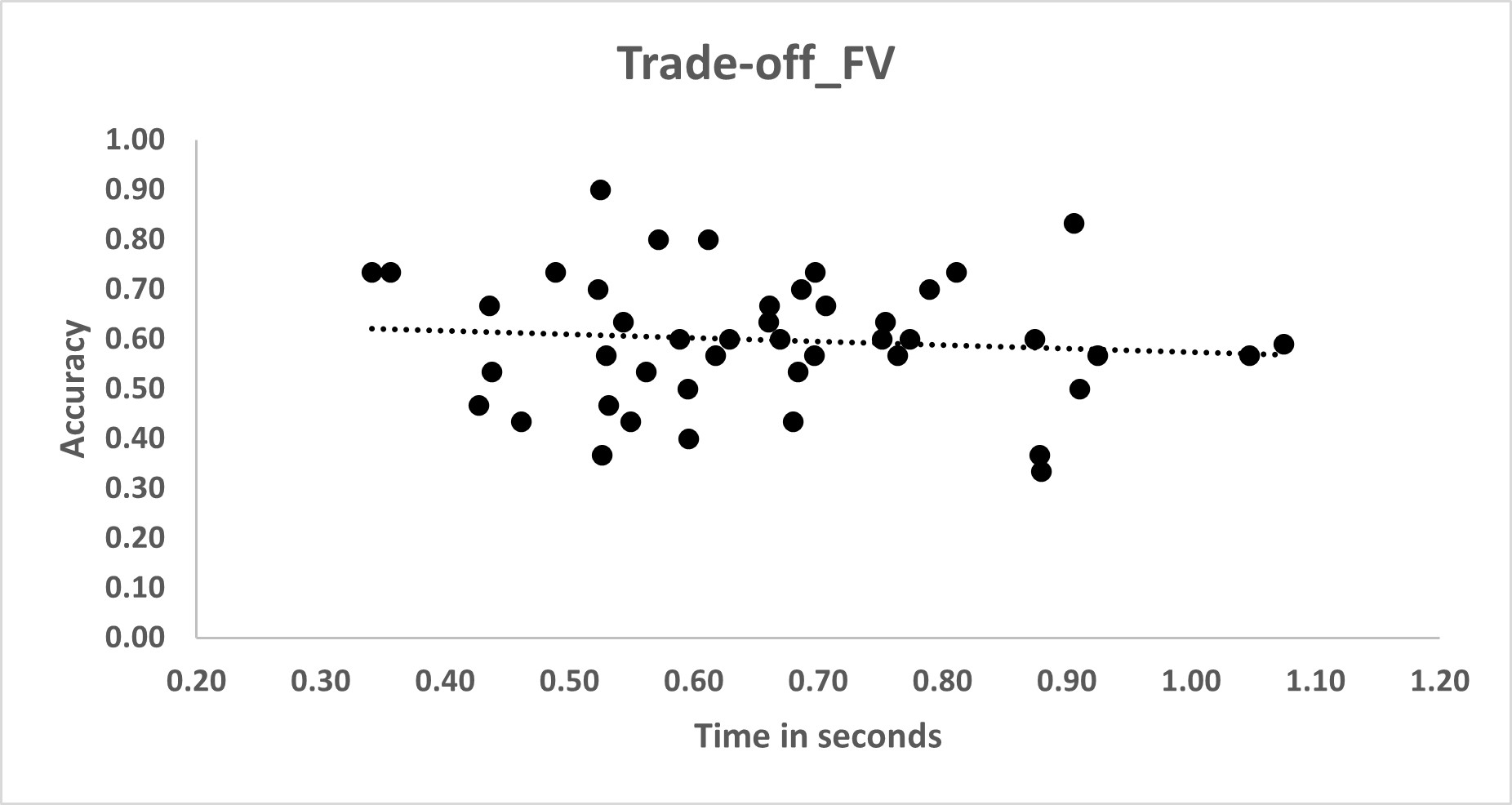


Figure S2. Trade-off in FV condition


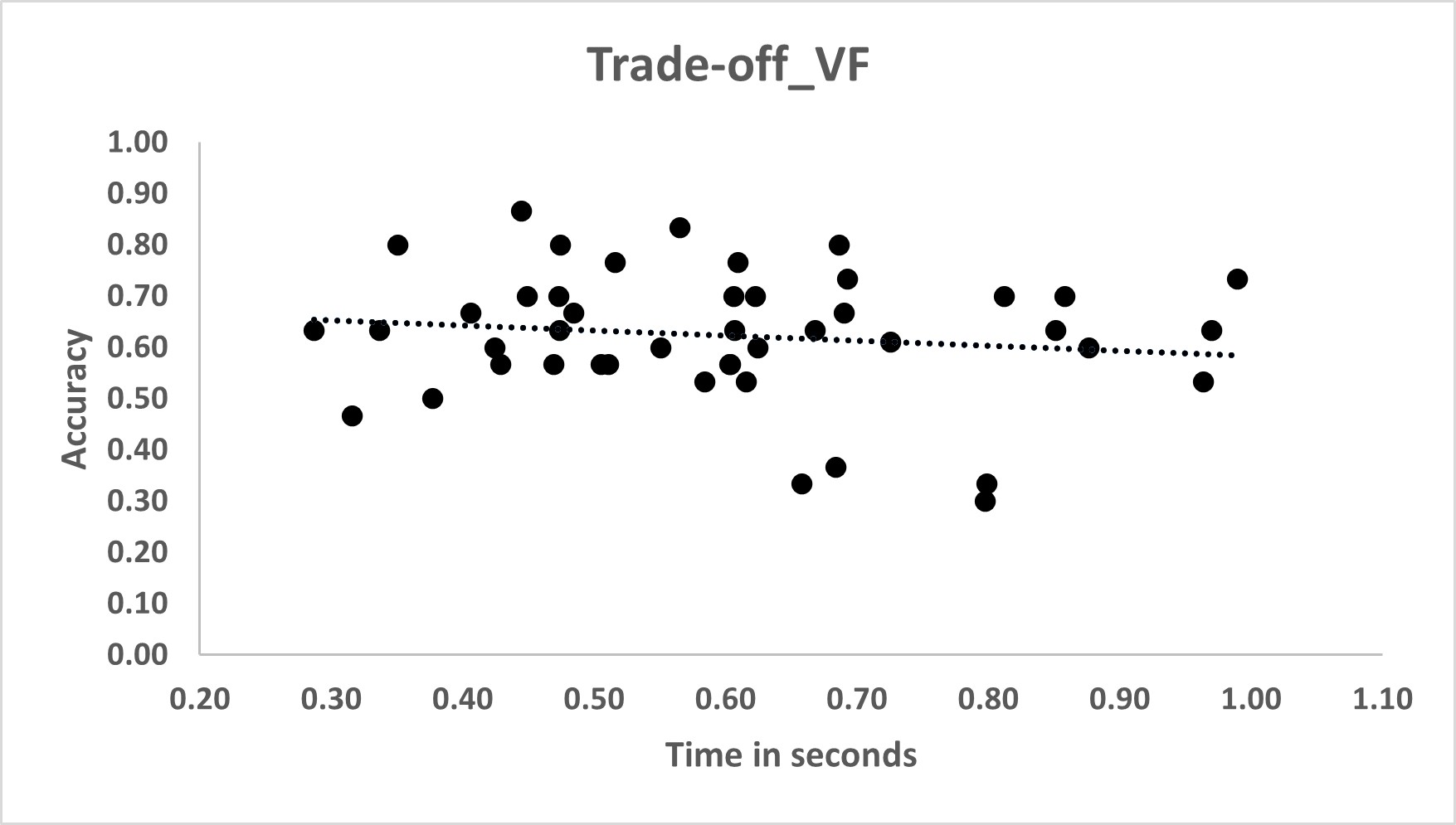


Figure S3. Trade-off in VF condition


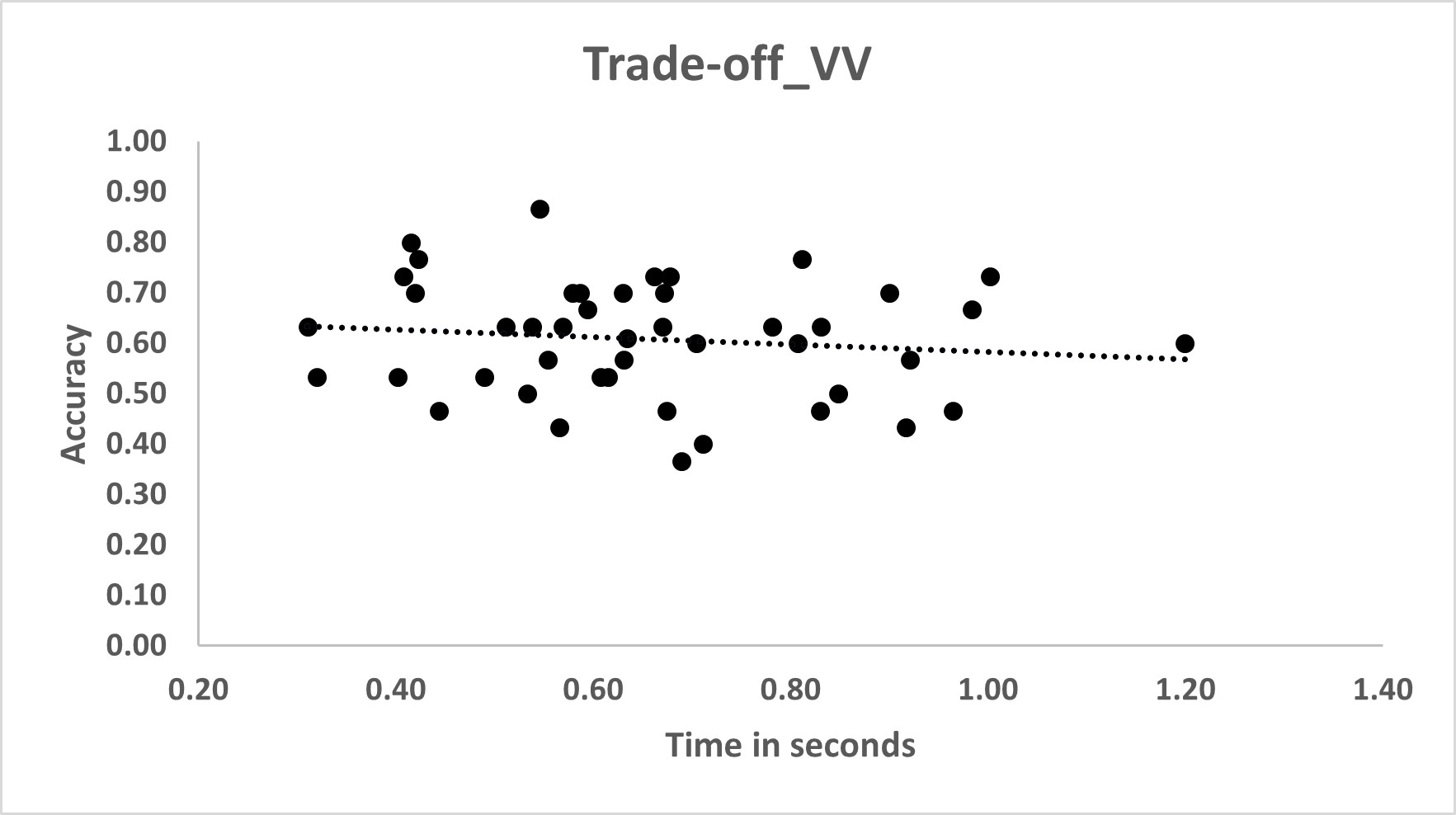


Figure S4. Trade-off in VV condition

**
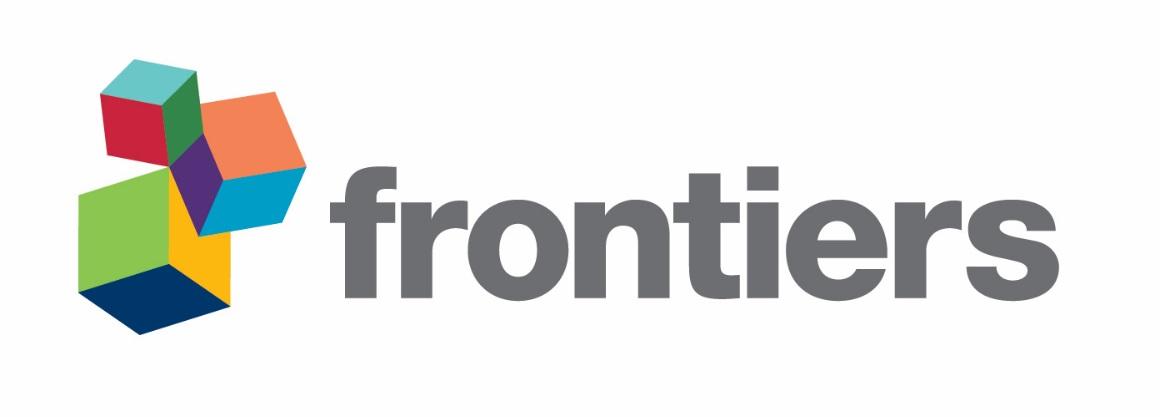
**
